# ICTV Virus Taxonomy Profile: Duplodnaviria 2025

**DOI:** 10.1099/jgv.0.002139

**Published:** 2025-09-02

**Authors:** Evelien M. Adriaenssens, Ryan Cook, Valerian Dolja, Eugene V. Koonin, Mart Krupovic, Jens H. Kuhn, Cédric Lood, Alejandro Reyes Muñoz, Dann Turner, Arvind Varsani, Paola K. Vaz, Thomas Waltzek, Yuri I. Wolf, Natalya Yutin, F. Murilo Zerbini

**Affiliations:** 1 Quadram Institute Bioscience, Norwich, UK; 2 Centre for Microbial Interactions, Norwich Research Park, Norwich NR4 7UG, UK; 3 Department of Botany and Plant Pathology, Oregon State University, Corvallis, OR 97330, USA; 4 Division of Intramural Research, National Library of Medicine, National Institutes of Health, Bethesda, MA, USA; 5 Institut Pasteur, Université Paris Cité, CNRS UMR6047, Cell Biology and Virology of Archaea Unit, 75015 Paris, France; 6 NIH/NIAID/IRF-Frederick, Fort Detrick, Frederick, MD 21702, USA; 7 Department of Biology, University of Oxford, Oxford, UK; 8 Universidad de los Andes, Bogotá, 111711, Colombia; 9 School of Applied Sciences, College of Health, Science and Society, University of the West of England, Bristol, Frenchay Campus, Coldharbour Lane, Bristol BS16 1QY, UK; 10 Biodesign Center for Fundamental and Applied Microbiomics, Center for Evolution and Medicine, School of Life Sciences, Arizona State University, Tempe, AZ 85042, Arizona, USA; 11 Melbourne Veterinary School, University of Melbourne, Victoria, Australia; 12 Washington Animal Disease Diagnostic Laboratory, Washington State University, Pullman, WA 99163, Washington, USA; 13 Department of Veterinary Microbiology and Pathology, Washington State University, Pullman, WA 99163, Washington, USA; 14 Dep. de Fitopatologia, UFVicosa, Viçosa, MG 36750-900, Brazil

**Keywords:** *Caudoviricetes*, double-stranded DNA viruses, *Duplodnaviria*, *Herviviricetes*, HK97-fold major capsid protein, ICTV Report, *Peploviricota*, *Uroviricota*, Virus Taxonomy

## Abstract

The realm *Duplodnaviria* includes viruses of archaea, bacteria and eukaryotes, with linear dsDNA genomes. Duplodnavirians share a distinct morphogenetic module of four hallmark genes encoding the HK97-fold major capsid protein, a genome packaging ATPase-nuclease (large terminase subunit), a portal protein and a capsid maturation protease. This is a summary of the International Committee on Taxonomy of Viruses (ICTV) Report on the realm *Duplodnaviria*, which is available at ictv.global/report/duplodnaviria.

**Table:** 

**Table 1** Characteristics of members of the realm *Duplodnaviria*	
Examples	Escherichia phage T4 (AF158101), species *Tequatrovirus T4*; Haloferax tailed virus 1 (MG550112), species *Retbasiphovirus hantatum*; pseudorabies virus (JF797218), species *Varicellovirus suidalpha1*
Virion	Icosahedral capsids, with or without an envelope, with (bacterial and archaeal viruses) or without (eukaryotic viruses) helical tails
Genome	A single linear segment of dsDNA of 11.6–660 kbp. Reiterated sequences are common. Terminase-type DNA packaging. Modular and scattered gene organization
Replication	Rolling concatemeric, replicative transposition or protein-primed DNA replication using virus- or host-encoded enzymes. DNA-templated transcription by host- or virus-encoded RNA polymerase may be temporally co-ordinated.
Translation	Some mRNAs are spliced
Host range	Bacteria, archaea, eukaryotes
Taxonomy	The realm includes 1 kingdom, 2 phyla, 2 classes, >10 orders, >100 families, >130 subfamilies, >1700 genera and >5900 species

## Virion

Particles of viruses of the class *Herviviricetes* (phylum *Peploviricota,* eukaryotic hosts) are 150–200 nm in diameter and are pleomorphic, mostly spherical, with a glycoprotein-containing lipid envelope that encloses a tegument and an icosahedral capsid.

Particles of viruses of the class *Caudoviricetes* (phylum *Uroviricota*, bacterial or archaeal hosts) are head-tailed and do not have an envelope[1,2]. The head is icosahedral and may be isometric or prolate (head diameter 40–200 nm). The tail is typically 10–350 nm in length but can be around 800 nm in some bacterial viruses [3].

The protein composition of mature particles varies greatly among viruses of different families. Only herviviricete particles contain lipids. Carbohydrates have been reported for herviviricete virions [4] and certain caudoviricetes infecting mycobacteria [5].

## Genome

All duplodnavirians have linear dsDNA genomes when packaged in the particle. Genome lengths are 108.4–322.3 kbp for herviviricetes and 11.6–>660 kbp for caudoviricetes. Depending on the replication and packaging mechanisms, genomes can have defined termini with reiterated sequences (direct or inverted repeats), cohesive 3′- or 5′-overhangs, or circularly permuted genome architectures [6]. Genes with associated functions are typically clustered, with conserved, essential genes localized internally/centrally in the genome; scattered gene arrangements also occur.

## Replication

Duplodnavirians with longer genomes usually encode their own DNA polymerases [7]. Herpesvirals uniformly encode family B DNA polymerases and several other replication proteins, suggesting semiautonomous genome replication [7]. Caudoviricetes can encode DNA polymerases of families A, B (both RNA- and protein-primed) and C [8]. Transposable phages (e.g. coliphage Mu, species *Muvirus mu*) use a different replication mechanism called replicative transposition, during which the viral genome is copied at various sites of the bacterial chromosome before transcription of structural proteins and packaging.

## Taxonomy

**Fig. 1.**
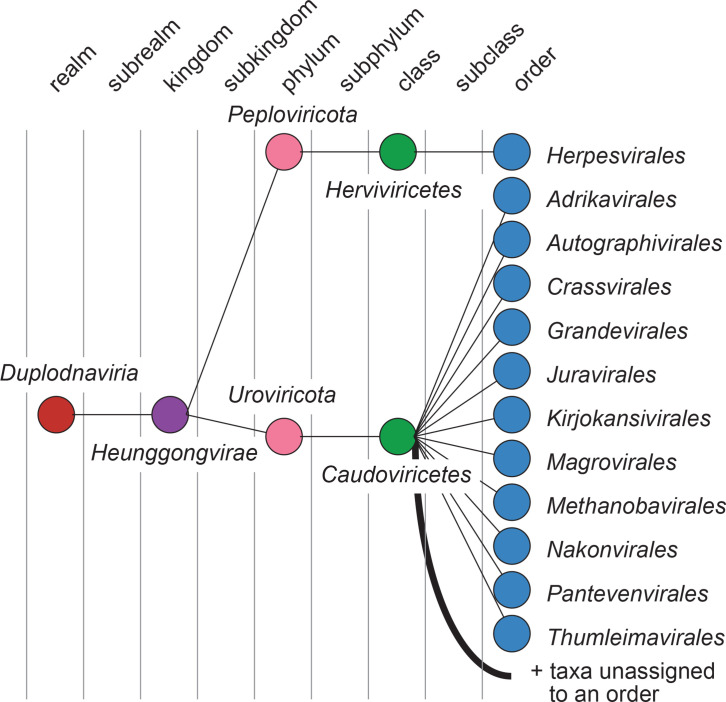
Current taxonomy of the realm *Duplodnaviria*.

Current taxonomy: ictv.global/taxonomy. The realm *Duplodnaviria* was established in 2020 (Master Species List #35) (Fig. 1). Members have a dsDNA genome encoding a morphogenetic module consisting of the major capsid protein with the HK97 structural fold, a portal protein, the terminase complex and a capsid maturation protease. The module is distinct from that encoded by other known dsDNA viruses, including those in the realm *Varidnaviria*.

## Resources

Full ICTV Report on the realm *Duplodnaviria*: ictv.global/report/duplodnaviria.
